# Relationship between polypharmacy and activities of daily living or in-hospital falls in elderly patients hospitalized for proximal femoral fracture: A retrospective cohort study

**DOI:** 10.1097/MD.0000000000044481

**Published:** 2025-09-12

**Authors:** Eisuke Takeshima, Akira Kimura

**Affiliations:** a Department of Physical Therapy, Faculty of Medical and Health Sciences, Hokuriku University, Kanazawa, Ishikawa, Japan; b Department of Health Science, Graduate School of Health Sciences, Gunma PAZ University, Takasaki, Gunma, Japan.

**Keywords:** activities of daily living, elderly people, functional independence measure, polypharmacy, proximal femoral fracture

## Abstract

Polypharmacy is a risk factor for falls and decreased functional ability in older adults; however, its age-specific impact during rehabilitation remains unclear. This study was conducted at Tsurugi Hospital, located in Ishikawa, Japan. This study aimed to investigate the association between polypharmacy and outcomes such as in-hospital falls and activities of daily living at discharge in elderly patients hospitalized for proximal femoral fractures, focusing on age-group differences. This retrospective cohort study was conducted at a comprehensive rehabilitation ward. Patients aged ≥75 years admitted between April 2020 and March 2022 were included in the study. Participants were stratified into 2 age groups (75–89 and ≥90 years). Polypharmacy was assessed using 2 definitions: ≥5 medications (conventional) and age group-specific median cutoffs. The functional independence measure (FIM) scores and fall incidence were analyzed using appropriate statistical methods, including multiple regression and Bonferroni correction. Eighty-six patients (mean age 89.3 ± 5.2 years) were analyzed. In the 75–89 years age group, polypharmacy defined as taking ≥8 medications at discharge was significantly associated with lower cognitive FIM scores (median: 23 vs 32; *r* = −0.384, *P* = .016). Subscale analyses revealed significant negative associations in comprehension (*r* = −0.463, *P* = .004), problem-solving (*r* = −0.325, *P* = .047), and memory (*r* = −0.360, *P* = .026). In the same age group, multiple regression analysis further confirmed that taking ≥9 medications at discharge was an independent predictor of lower cognitive FIM scores (*β* = −0.466, *P* = .003). In contrast, in the ≥90 years group, cognitive FIM scores were significantly negatively predicted by the presence of dementia (*β* = −0.376, *P* = .005). In the overall sample, body mass index (*β* = 0.284, *P* = .007) and shorter postoperative hospitalization duration (*β* = −0.274, *P* = .010) were also associated with better cognitive FIM scores. The relationship between polypharmacy and functional outcomes varied by age group. These findings highlight the need for individualized discharge planning and medication management based on patient age and background.

## 1. Introduction

Japan is one of the world’s leading countries in terms of longevity.^[1]^ With an increasing aging population, the frequency of falls and fall-related fractures is also rising, leading to a growing number of older adults requiring long-term care.^[2]^ Proximal femoral fractures are common injuries resulting from falls in older adults and are known to have long-term effects on activities of daily living (ADL) and life prognosis.^[3,4]^

Older adults often have multiple comorbidities that increase the number of medications they take, and polypharmacy has been recognized as a risk factor for falls and a decline in ADL.^[5,6]^ Polypharmacy is generally defined as the prescription of 5 or 6 oral medications.^[7–9]^ Prescriptions of ≥10 oral medications are defined as hyper-polypharmacy.^[10–12]^ During hospitalization, medications are often added for postoperative pain management, infection prevention, and delirium control, contributing to increased risk of polypharmacy and hyper-polypharmacy.^[13–15]^ Thus, drug interactions and side effects may lead to decreased attention, orthostatic hypotension, and unsteadiness, potentially causing falls and a decline in physical and cognitive function.^[16–18]^ The criteria for polypharmacy in hospitalized older adults may differ from those used for community-dwelling older adults; this remains an issue that warrants further investigation. Moreover, the effects of drug interactions and side effects associated with polypharmacy have been reported to vary depending on the age and underlying health conditions.^[19–21]^ It is thought that differences in drug sensitivity and recovery of ADL are more likely to occur in adults in the elderly (75–89 years) and very elderly (≥90 years) age groups. Therefore, examining the relationship between polypharmacy and outcomes such as falls and ADL by age group may contribute to the individualization of discharge planning and rehabilitation strategies.

Several studies have reported falls and adverse drug events associated with polypharmacy among older adults in acute care hospitals and long-term care facilities. Drug-related adverse events occurred in approximately 5% of elderly patients admitted to acute care facilities in Japan, and polypharmacy was significantly associated with these drug-related adverse events.^[22]^ Among elderly patients who experienced falls during hospitalization in acute care hospitals, those who took tricyclic antidepressants, diuretics, and narcotics had a 3.36-fold, 1.83-fold, and 2.09-fold higher risk of falling, respectively.^[23]^ Also, patients in long-term facilities frequently suffer from many adverse events.^[24]^ Polypharmacy was found to be common among elderly Chinese patients during hospitalization and was an independent risk factor for falls within 1 year.^[25]^ However, the effect of polypharmacy on older adults during the transitional period, when physical function and ADL undergo significant changes, has not been sufficiently investigated. This study aimed to investigate the association between polypharmacy at discharge and in-hospital falls and ADL at discharge in elderly patients with proximal femoral fractures admitted to a comprehensive rehabilitation ward. The analysis was conducted by age group based on the hypothesis that the impact of polypharmacy on discharge outcomes may differ depending on age.

## 2. Methods

### 2.1. Study design and participants

This was a retrospective cohort study at a single institution. This study included elderly patients who were admitted to the comprehensive rehabilitation ward of Tsurugi Hospital between April 2020 and March 2022. The inclusion criteria were: age ≥75 years; admitted with a diagnosis of proximal femoral fracture; and able to undergo functional independence measure (FIM) assessment at both admission and discharge. The exclusion criteria were as follows: age <75 years and missing data. Patients with comorbidities such as diabetes mellitus, cardiovascular disease, or dementia were not excluded and were analyzed accordingly. Medical histories are shown in Table 1.

**Table 1:** Characteristics of the total participants and age-based subgroups (75–89 years and 90 years and above).

	Total (n = 86)	Aged 75–89 (n = 39)	Aged 90 and above (n = 47)	*P*-value	Effect size
Age (yr)	89.3 ± 5.2	85 (83–87)	92 (91–94)	<.001‡	*r* = 0.860
Sex, male (%)	14 (16.3)	7 (17.9)	7 (14.9)	.702*	*V* = 0.041
BMI (kg/m^2^)	20.8 ± 3.8	20.8 ± 4.2	20.8 ± 3.6	.967§	*d* = 0.009
Postoperative to hospitalization (d)	17 (16–23)	17 (13–24)	18.7 ± 6.8	.997‡	*r* < 0.001
Length of stay (d)	54 (33–73)	53.4 ± 22.0	56 (32–71)	.862‡	*r* = −0.019
Fallers, n (%)	22 (25.6)	10 (25.6)	12 (25.5)	.991*	*V* = 0.001
Medications					
Oral medications, types (admission)	7 (4–8)	8 (4–9)	5.8 ± 2.6	.017‡	*r* = −0.257
Oral medications, types (discharge)	6.0 ± 2.9	6.9 ± 2.9	5.3 ± 2.7	.007§	*d* = 0.595
BDZs or non-BDZs, n (%) (admission)	7 (8.1)	3 (7.7)	4 (8.5)	1.000†	*V* = 0.015
BDZs or non-BDZs, n (%) (discharge)	8 (9.3)	4 (10.3)	4 (8.5)	1.000†	*V* = 0.030
α or αβ-blockers, n (%) (admission)	4 (4.7)	2 (5.1)	2 (4.3)	1.000†	*V* = 0.021
α or αβ-blockers, n (%) (discharge)	13 (15.1)	5 (12.8)	8 (17.0)	.588*	*V* = 0.058
Loop diuretics, n (%) (admission)	7 (8.1)	3 (7.7)	4 (8.5)	1.000†	*V* = 0.015
Loop diuretics, n (%) (discharge)	15 (17.4)	7 (17.9)	8 (17.0)	.910*	*V* = 0.016
Medical history					
Cerebrovascular disorder, n (%)	13 (15.1)	6 (15.4)	7 (14.9)	.950*	*V* = 0.007
Fracture, n (%)	35 (40.7)	14 (35.9)	21 (44.7)	.409*	*V* = 0.089
Dementia, n (%)	13 (15.1)	7 (17.9)	6 (12.8)	.504*	*V* = 0.072
Diabetes mellitus, n (%)	23 (26.7)	11 (28.2)	12 (25.5)	.780*	*V* = 0.030
Cardiovascular disease, n (%)	26 (30.2)	11 (28.2)	15 (31.9)	.709*	*V* = 0.040
Depression/psychosis, n (%)	1 (1.2)	1 (2.6)	0 (0.0)	.453†	*V* = 0.119

Data are presented as mean ± SD or median (IQR), according to data distribution assessed by the Shapiro–Wilk test. Categorical variables are shown as number (%). Effect size was calculated using Cohen *d* for *t*-test, rank biserial correlation *r* for Mann–Whitney *U* test, and Cramér *V* for categorical variables.

BDZs = benzodiazepines, BMI = body mass index, IQR = interquartile range, SD = standard deviation.

* *P*-value from chi-square test.

† *P*-value from Fisher exact test.

‡ *P*-value from Mann–Whitney *U* test.

§ *P*-value from independent *t*-test.

### 2.2. Position of this study and sample size

Based on the study design, this study was positioned as a hypothesis-generating exploratory analysis. The study population was limited to patients admitted during the study period who met the inclusion criteria. Because in-hospital falls are relatively rare, and due to restrictions in daily activities among older adults during the coronavirus disease 2019 pandemic, it was difficult to collect additional cases. In interpreting the statistical analyses, careful consideration was given not only to *P*-values but also to effect sizes, which were independent of the sample size.

### 2.3. Ethics

This study was approved by the Ethics Committees of Tsurugi Hospital (Approval no. 5-1) and the Graduate School of Gunma Paz University. This study was registered in the clinical trial registry (University Hospital Medical Information Network: UMIN000054230). As this study did not involve any invasive procedures or interventions with the participants, written informed consent was not obtained. Instead, information regarding the study’s objectives, methods, and data protection policy was made publicly available via posters displayed at the hospital to provide an opportunity for opt-out. Careful consideration was given to ensure that no disadvantage was imposed on the participants, and all analyses were conducted using anonymized data to protect individual identities.

### 2.4. Demographic data

Information on the participants’ age, sex, body mass index (BMI), number of days from surgery to transfer, length of hospital stay, and medical history were collected from electronic medical records.

### 2.5. Medication information at admission and discharge, and evaluation of polypharmacy

Information on oral medications at admission and discharge was collected from electronic medical records, focusing only on regularly prescribed oral medications. The data included the type, dosage, and pharmacological class of each medication. Although there is no universally accepted definition of polypharmacy, previous studies in Japan and abroad have often considered the use of 5 to ≥6 medications as a general benchmark.^[7–9,26,27]^ In this study, polypharmacy was defined as the use of ≥5 oral medications based on a previous study that examined the association between polypharmacy and falls in Japanese outpatients.^[28]^ However, considering that the characteristics of hospitalized older adults may differ from those of outpatient populations, we also explored an alternative criterion, using the median number of prescribed medications at admission and discharge within our study population as a potential cutoff value.

### 2.6. Assessment of in-hospital falls

Based on the World Health Organization definition, “Falls are events that result in a person coming to rest inadvertently on the ground, floor, or other lower levels.”^[29]^ The presence of falls was determined by reviewing electronic medical records and observation notes recorded by the medical staff. If a fall was reported once during the hospital stay, the patient was classified as having experienced a fall.

### 2.7. Assessment of ADL

ADL was assessed using the FIM.^[30,31]^ The FIM consists of 18 items, including 13 motor and 5 cognitive items. The 13 motor subitems included eating, grooming, bathing, dressing the upper body, dressing the lower body, toileting, bladder management, bowel management, transfers to/from bed/chair/wheelchair, transfers to/from the toilet, transfers to/from the tub or shower, walking or wheelchair mobility, and stair climbing. The 5 cognitive subitems are comprehension, expression, social interaction, problem-solving, and memory. Each item is scored on a scale of 1 to 7, with a total score ranging from 18 to 126, indicating the level of independence in ADL. A higher total score indicated greater independence in ADL and has been reported to be associated with fall risk and the level of independence in daily life after discharge.^[32,33]^ The intraclass correlation coefficients for the FIM were reported as 0.96 for the total score, 0.96 for the motor domain total score, and 0.91 for the cognitive domain total score.^[34]^ It has been reported that the FIM is useful as a functional assessment scale for elderly people aged 80 years or older, provided that certain precautions are taken.^[35]^ The FIM assessment was conducted by the same evaluators (physical therapists and occupational therapists with 2–20 years of experience) within 1 week of admission to Tsurugi Hospital and 1 week before discharge. The evaluation results of therapists with less experience were reviewed by experienced therapists.

### 2.8. Statistical analyses

The normality of the collected data was assessed using the Shapiro–Wilk test. For variables with a normal distribution, data were presented as mean ± standard deviation. Data were expressed as median (interquartile range) for variables that did not follow a normal distribution. Quantitative data were analyzed using the independent *t*-test or Mann–Whitney *U* test, whereas categorical data were compared using the chi-square test or Fisher exact test. The significance level for the 2-group comparisons was set at 5%. The interpretation was not based solely on *P*-values; effect sizes that were not influenced by the sample size were also calculated and considered. Effect sizes were reported as Cohen *d* for the *t*-test, rank biserial correlation coefficient *r* for the Mann–Whitney *U* test, and Cramér *V* for categorical variables.

Participants were stratified by age into 2 groups based on the criteria for older adults proposed by the Japan Gerontological Society and the Japan Geriatrics Society: 75–89 years and ≥90.^[36]^ Within each age group, the participants were further divided into 2 subgroups based on the presence or absence of falls during hospitalization, and analyses were conducted to examine the relationship between the number of prescribed medications and medications known to be associated with falls. Additionally, the participants were categorized into 2 groups based on the presence or absence of polypharmacy at discharge, and analyses were performed to assess the associations between demographic characteristics and FIM scores at discharge.

Multiple regression analysis was performed with FIM scores at discharge as the dependent variable. Independent variables included age, sex (male = 1, female = 0), BMI, the number of days from surgery to transfer, length of hospital stay, the number of prescribed medications at admission and discharge, presence of 5 to 10 medications based on various cutoff values (≥cutoff = 1, <cutoff = 0), and history of comorbidities (cerebrovascular disease, fractures, and dementia). Variables were selected using a stepwise method. The multiple regression analysis was carried out 9 times, combining 3 dependent variables (FIM total, FIM motor, and FIM cognitive) with 3 participant groups (total sample, ages 75–89, and ages 90 and above). Bonferroni correction was applied to adjust for multiple comparisons, setting the significance level at 0.05/9 = 0.0056 (approximately 0.56%). All statistical analyses were performed using SPSS version 29.0.2.0 (Windows: IBM, Armonk).

## 3. Results

**Figure 1.**
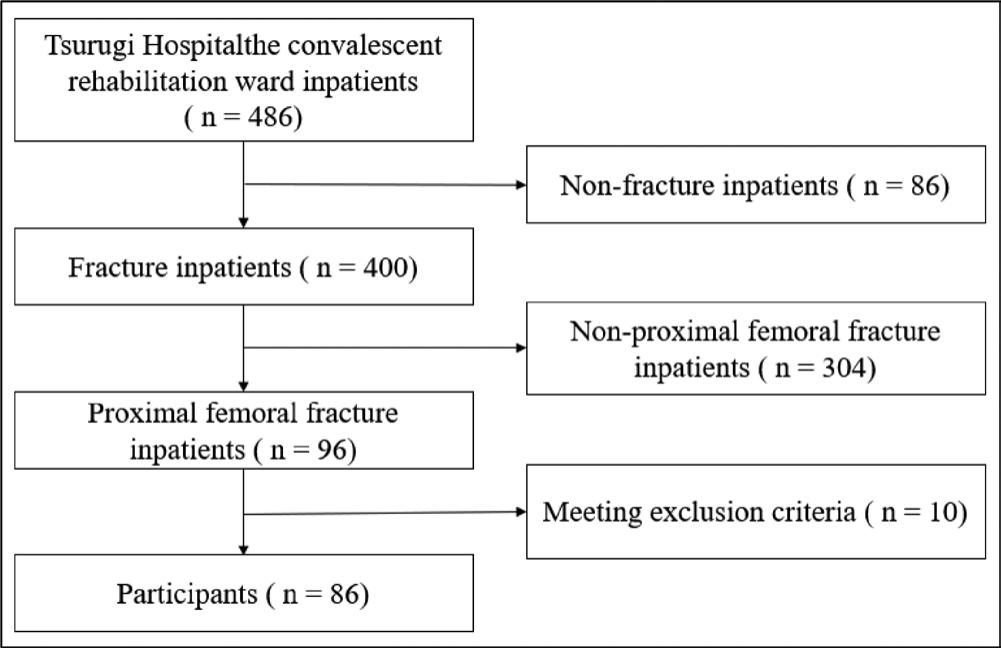
Flowchart of participant selection.

A flowchart of the study is shown in Figure 1. Ninety-six patients with proximal femoral fractures were admitted to our hospital during the study period. After excluding 10 patients ˂75 years of age based on the exclusion criteria, 86 patients (14 men and 72 women; mean age 89.3 ± 5.2 years) were included in the analysis. No missing values were observed in the data collected.

### 3.1. Participant characteristics by age group

Table 1 presents the characteristics of all participants, stratified by age group (75–89 years and ≥90 years). The number of regularly prescribed oral medications at both admission and discharge was significantly higher in the 75–89 years age group (admission: *P* = .017, *r* = −0.257; discharge: *P* = .007, *d* = 0.595). No other significant differences were observed between the groups except for age (*P* > .05).

### 3.2. Comparison of medication use and medical history by fall status

Table 2 compares medication use and medical history between fallers and non-fallers, in the overall cohort and within each age group (75–89 years and ≥90). No significant differences were found between the fall and non-fall groups regarding the number of oral medications used, the use of specific medications associated with falls, or medical histories (*P* > .05). However, at discharge, patients taking diabetic medications tended to be more common among the non-fallers, although this trend was not statistically significant (total: *V* = 0.197, *P* = .068).

**Table 2:** Comparison of medication use and medical history between fallers and non-fallers in the total participants and by age group (75–89 years and 90 years and above).

	Total faller (n = 22)	Non-faller (n = 64)	*P*-value	Effect size	Aged 75–89 Faller (n = 10)	Non-faller (n = 29)	*P*-value	Effect size	Aged 90 and above Faller (n = 12)	Non-faller (n = 35)	*P*-value	Effect size
Medications, types (admission)	6.4 ± 3.2	6.2 ± 2.7	.738§	*d* = −0.083	8 (6–10)	7 (4–9)	.418‡	*r* = 0.135	5.5 ± 3.1	5.6 ± 2.5	.911§	*d* = 0.038
Medications, types (discharge)	6.1 ± 3.2	6.0 ± 2.8	.899§	*d* = −0.031	8 (6–10)	7 (4–9)	.475‡	*r* = 0.120	5.1 ± 3.1	5.3 ± 2.6	.775§	*d* = 0.096
BDZs or non-BDZs, n (%) (admission)	1 (4.5)	6 (9.4)	.672†	*V* = 0.077	0 (0)	3 (10.3)	.556†	*V* = 0.170	1 (8.3)	3 (8.6)	1.000†	*V* = 0.004
BDZs or non-BDZs, n (%) (discharge)	1 (4.5)	7 (10.9)	.674†	*V* = 0.096	0 (0)	4 (13.8)	.556†	*V* = 0.199	1 (8.3)	3 (8.6)	1.000†	*V* = 0.004
α or αβ-blockers, n (%) (admission)	0 (0)	4 (6.3)	.568†	*V* = 0.129	0 (0)	2 (6.9)	1.000†	*V* = 0.137	0 (0)	2 (5.7)	1.000†	*V* = 0.123
α or αβ-blockers, n (%) (discharge)	4 (18.2)	9 (14.1)	.732†	*V* = 0.050	1 (10.0)	4 (13.8)	1.000†	*V* = 0.050	3 (25.0)	5 (14.3)	.403†	*V* = 0.124
Loop diuretics, n (%) (admission)	1 (4.5)	6 (9.4)	.672†	*V* = 0.077	0 (0)	3 (10.3)	.556†	*V* = 0.170	1 (8.3)	3 (8.6)	1.000†	*V* = 0.004
Loop diuretics, n (%) (discharge)	3 (13.6)	12 (18.8)	.750†	*V* = 0.059	1 (10.0)	6 (20.7)	.653†	*V* = 0.122	2 (16.7)	6 (17.1)	1.000†	*V* = 0.006
Diabetic drugs, n (%) (admission)	0 (0)	5 (7.8)	.322†	*V* = 0.146	0 (0)	2 (6.9)	1.000†	*V* = 0.137	0 (0)	3 (8.6)	.560†	*V* = 0.153
Diabetic drugs, n (%) (discharge)	2 (9.1)	18 (28.1)	.068*	*V* = 0.197	1 (10.0)	9 (31.0)	.402†	*V* = 0.210	1 (8.3)	9 (25.7)	.414†	*V* = 0.185
Cerebrovascular disorder, n (%)	3 (13.6)	10 (15.6)	1.000†	*V* = 0.024	1 (10.0)	6 (20.7)	.653†	*V* = 0.122	2 (16.7)	4 (11.4)	.637†	*V* = 0.068
Fracture, n (%)	8 (36.4)	25 (39.1)	.822*	*V* = 0.024	4 (40.0)	9 (31.0)	.704†	*V* = 0.083	4 (33.3)	16 (45.7)	.454*	*V* = 0.109
Dementia, n (%)	3 (13.6)	10 (15.6)	1.000†	*V* = 0.024	2 (20.0)	5 (17.2)	1.000†	*V* = 0.031	1 (8.3)	5 (14.3)	1.000†	*V* = 0.078

Data are presented as mean ± SD, median (IQR), or number (%), as appropriate. The choice between mean or median was based on the results of the Shapiro–Wilk test for normality. Effect size was calculated using Cohen *d* for *t*-test, rank biserial correlation *r* for Mann–Whitney *U* test, and Cramér *V* for categorical variables.

BDZs = benzodiazepines, IQR = interquartile range, SD = standard deviation.

* *P*-value from chi-square test.

† *P*-value from Fisher exact test.

‡ *P*-value from Mann–Whitney *U* test.

§ *P*-value from independent *t*-test.

### 3.3. Association between polypharmacy and falls across age groups

Table 3 compares fall incidence based on thresholds for polypharmacy at admission and discharge, stratified by age group (75–89 years and ≥90 years). Cutoff values ranging from 5 to 10 medications were applied to the number of regularly prescribed oral medications. Comparisons between fallers and non-fallers in each age group revealed no significant differences (*P* > .05). The median number of medications used to divide each age group into 2 equal parts was 8 for the 75–89 years age group and 6 for the ≥90 years.

**Table 3:** Comparison of fall incidence according to polypharmacy thresholds at admission and discharge, by age group (75–89 years and 90 years and above).

	Total (n = 86)	Aged 75–89 Faller (n = 10)	Non-faller (n = 29)	*P*-value	Effect size	Aged 90 and above Faller (n = 12)	Non-faller (n = 35)	*P*-value	Effect size
5 or more medications, n (%) (admission)	58 (67.4)	8 (80.0)	20 (69.0)	.693†	*V* = 0.107	8 (66.7)	22 (62.9)	1.000†	*V* = 0.035
5 or more medications, n (%) (discharge)	68 (79.1)	8 (80.0)	25 (86.2)	.636†	*V* = 0.075	9 (75.0)	26 (74.3)	1.000†	*V* = 0.007
6 or more medications, n (%) (admission)	50 (58.1)	8 (80.0)	19 (65.5)	.693†	*V* = 0.137	5 (41.7)	18 (51.4)	.559*	*V* = 0.085
6 or more medications, n (%) (discharge)	49 (57.0)	8 (80.0)	19 (65.5)	.693†	*V* = 0.137	4 (33.3)	18 (51.4)	.331*	*V* = 0.158
7 or more medications, n (%) (admission)	46 (53.5)	7 (70.0)	19 (65.5)	1.000†	*V* = 0.042	5 (41.7)	15 (42.9)	1.000†	*V* = 0.010
7 or more medications, n (%) (discharge)	42 (48.8)	7 (70.0)	19 (65.5)	1.000†	*V* = 0.042	4 (33.3)	12 (34.3)	1.000†	*V* = 0.009
8 or more medications, n (%) (admission)	32 (37.2)	7 (70.0)	13 (44.8)	.273†	*V* = 0.220	3 (25.0)	9 (25.7)	1.000†	*V* = 0.007
8 or more medications, n (%) (discharge)	29 (33.7)	7 (70.0)	12 (41.4)	.155†	*V* = 0.250	2 (16.7)	8 (22.9)	1.000†	*V* = 0.066
9 or more medications, n (%) (admission)	19 (22.1)	5 (50.0)	8 (27.6)	.253†	*V* = 0.208	2 (16.7)	4 (11.4)	.637†	*V* = 0.068
9 or more medications, n (%) (discharge)	17 (19.8)	4 (40.0)	8 (27.6)	.693†	*V* = 0.117	2 (16.7)	3 (8.6)	.590†	*V* = 0.114
10 or more medications, n (%) (admission)	12 (14.0)	4 (40.0)	6 (20.7)	.244†	*V* = 0.193	1 (8.3)	1 (2.9)	.450†	*V* = 0.118
10 or more medications, n (%) (discharge)	10 (11.6)	3 (30.0)	5 (17.2)	.399†	*V* = 0.138	1 (8.3)	1 (2.9)	.450†	*V* = 0.118

Values are presented as number (%).

* *P*-value from chi-square test.

† *P*-value from Fisher exact test.

### 3.4. Discharge characteristics and FIM scores by conventional polypharmacy definition

Table 4 compares patient characteristics and FIM scores at discharge based on the presence or absence of polypharmacy, defined as the use of ≥5 medications. Within each age group, the participants were divided into 2 groups according to this cutoff value, as established in previous studies. However, no significant differences were observed between the groups (*P* > .05).

**Table 4:** Comparison of patient characteristics and FIM scores at discharge between patients with and without polypharmacy, defined as the use of 5 or more medications.

	Aged 75–89 Polypharmacy yes (n = 33)	No (n = 6)	*P*-value	Effect size	Aged 90 and above Polypharmacy yes (n = 35)	No (n = 12)	*P*-value	Effect size
Age (yr)	84.6 ± 3.2	84.2 ± 3.0	.756†	*d* = −0.139	92 (91–94)	94 (92–96)	.260*	*r* = −0.164
Sex, male (%)	5 (15.2)	2 (33.3)	.290‡	*V* = 0.171	6 (17.1)	1 (8.3)	.659‡	*V* = 0.108
BMI (kg/m^2^)	21.2 ± 4.2	18.6 ± 3.4	.161†	*d* = −0.635	21.2 ± 3.6	19.4 ± 3.4	.133†	*d* = −0.511
Postoperative to hospitalization (d)	18 (13–26)	16 (15–17)	.988*	*r* = −0.003	19.0 ± 6.6	18.0 ± 7.5	.674†	*d* = −0.142
Length of stay (d)	52.3 ± 22.3	60.0 ± 21.3	.457†	*d* = 0.334	61 (34–71)	38 (24–57)	.341*	*r* = 0.139
FIM total	64.8 ± 21.5	66.0 ± 20.0	.896†	*d* = 0.058	103 (71 to 113)	76 (60–108)	.457*	*r* = 0.109
Motor items	39.0 ± 16.8	40.5 ± 13.5	.838†	*d* = 0.091	75 (52–80)	57 (35–77)	.428*	*r* = 0.116
Cognitive items	25.8 ± 7.2	25.5 ± 7.0	.936†	*d* = −0.036	27 (20–31)	24 (17–30)	.448*	*r* = 0.111
Eating	6 (5–6)	5 (5–7)	.286*	*r* = −0.179	7 (6–7)	6 (5–7)	.083*	*r* = 0.252
Grooming	5 (2–5)	4 (2–5)	.842*	*r* = −0.037	6 (5–7)	5 (4–6)	.353*	*r* = 0.135
Bathing	2 (1–3)	3 (2–4)	.158*	*r* = −0.239	5 (3–5)	4 (2–5)	.891*	*r* = 0.020
Dressing upper body	1 (1–5)	1 (1–1)	.914*	*r* = −0.020	7 (4–7)	6 (1–7)	.486*	*r* = 0.102
Dressing lower body	1 (1–2)	1 (1–1)	.818*	*r* = 0.045	7 (4–7)	6 (1–7)	.502*	*r* = 0.098
Toileting	3 (1–5)	3 (3–5)	.678*	*r* = −0.069	6 (5–7)	5 (2–6)	.326*	*r* = 0.143
Bladder control	5 (1–6)	5 (5–6)	.414*	*r* = −0.139	6 (4–7)	5 (3–6)	.429*	*r* = 0.115
Bowel control	6 (1–7)	5 (4–6)	.770*	*r* = −0.052	6 (5–7)	5 (4–6)	.360*	*r* = 0.133
Bed/chair/wheelchair	4 (3–5)	4 (4–5)	.890*	*r* = 0.023	6 (5–6)	6 (5–6)	.617*	*r* = 0.073
Toilet	4 (1–5)	4 (4–5)	.988*	*r* = −0.005	6 (5–6)	5 (5–6)	.392*	*r* = 0.125
Tub/shower	1 (1–1)	1 (1–1)	1.000*	*r* = 0	5 (1–5)	1 (1–5)	.193*	*r* = 0.190
Walking/wheelchair use	1 (1–4)	1 (1–1)	1.000*	*r* = 0.006	6 (1–6)	1 (1–6)	.139*	*r* = 0.216
Stairs	1 (1–1)	1 (1–1)	.678*	*r* = −0.255	5 (1–5)	5 (1–5)	.740*	*r* = −0.048
Comprehension	6 (4–7)	6 (6–7)	.286*	*r* = −0.178	6 (4–7)	6 (4–6)	.630*	*r* = 0.070
Expression	6 (4–7)	6 (6–7)	.770*	*r* = −0.053	7 (5–7)	7 (3–7)	.968*	*r* = 0.006
Social interaction	7 (6–7)	6 (6–7)	.450*	*r* = −0.137	7 (6–7)	7 (5–7)	.744*	*r* = 0.048
Problem solving	4 (3–6)	3 (2–6)	.432*	*r* = 0.132	4 (2–6)	3 (1–5)	.413*	*r* = 0.119
Memory	4 (3–6)	3 (2–6)	.914*	*r* = 0.018	5 (2–6)	3 (2–4)	.212*	*r* = 0.182

Data are presented as mean ± SD, median (IQR), or number (%), as appropriate. Polypharmacy was defined as the regular use of 5 or more medications at discharge. The choice between mean or median was based on the results of the Shapiro–Wilk test for normality. Effect size was calculated using Cohen *d* for *t*-test, rank biserial correlation *r* for Mann–Whitney *U* test, and Cramér *V* for categorical variables.

BMI = body mass index, FIM = functional independence measure, SD = standard deviation, IQR = interquartile range.

* *P*-value from Mann–Whitney *U* test.

† *P*-value from independent *t*-test.

‡ *P*-value from Fisher exact test.

### 3.5. Discharge characteristics and FIM scores by age-specific polypharmacy cutoffs

Table 5 compares patient characteristics and FIM scores at discharge based on the presence or absence of polypharmacy, as defined using age group-specific cutoff values based on the median number of prescribed medications. In the 75–89 years age group, where polypharmacy was defined as the use of ≥8 medications, a significant negative association was observed with the total FIM cognitive score (*r* = −0.384, *P* = .016), and with the subitems of comprehension (*r* = −0.463, *P* = .004), problem-solving (*r* = −0.325, *P* = .047), and memory (*r* = −0.360, *P* = .026). In the ≥90 years, where polypharmacy was defined as the use of ≥6 medications, significant positive associations were observed with sex (*V* = 0.326, *P* = .040), BMI (Cohen *d* = 0.608, *P* = .043), and the FIM subitem of memory (*r* = 0.287, *P* = .049).

**Table 5:** Comparison of patient characteristics and FIM scores at discharge between patients with and without polypharmacy, defined by a median-based cutoff.

	Aged 75–89 Polypharmacy yes (n = 19)	No (n = 20)	*P*-value	Effect size	Aged 90 and above Polypharmacy yes (n = 22)	No (n = 25)	*P*-value	Effect size
Age (yr)	84.8 ± 2.6	84.3 ± 3.7	.671†	*d* = −0.137	92 (90–94)	94 (92–95)	.221*	*r* = −0.178
Sex, male (%)	3 (15.8)	4 (20.0)	1.000‡	*V* = 0.055	6 (27.3)	1 (4.0)	.040‡	*V* = 0.326
BMI (kg/m^2^)	20.9 (17.7–22.1)	20.7 (17.3–24.0)	.588*	*r* = −0.090	21.9 ± 3.8	19.8 ± 3.1	.043†	*d* = −0.608
Postoperative to hospitalization (d)	17 (13–21)	17 (13–24)	.857*	*r* = 0.029	19.4 ± 5.5	18.1 ± 7.8	.522†	*d* = −0.189
Length of stay (d)	54.0 ± 25.2	52.8 ± 18.8	.866†	*d* = −0.054	55.6 ± 21.0	50.6 ± 24.5	.457†	*d* = −0.219
FIM total	98 (38–106)	111 (91–117)	.065*	*r* = −0.295	103 (73–114)	92 (62–110)	.354*	*r* = 0.135
Motor items	73 (19–80)	78 (64–83)	.224*	*r* = −0.196	75 (52–80)	68 (45–80)	.337*	*r* = 0.140
Cognitive items	23 (19–28)	32 (25–35)	.016*	*r* = −0.384	27 (21–34)	25 (17–31)	.375*	*r* = 0.129
Eating	6 (4–7)	7 (6–7)	.351*	*r* = −0.166	7 (6–7)	6 (5–7)	.119*	*r* = 0.228
Grooming	5 (1–7)	6 (5–7)	.411*	*r* = −0.141	7 (5–7)	5 (4–7)	.091*	*r* = 0.246
Bathing	4 (1–5)	5 (4–6)	.134*	*r* = −0.248	5 (3–6)	5 (2–5)	.410*	*r* = 0.120
Dressing upper body	6 (1–7)	7 (6–7)	.141*	*r* = −0.258	7 (5–7)	6 (3–7)	.175*	*r* = 0.198
Dressing lower body	6 (1–7)	6 (6–7)	.478*	*r* = −0.122	7 (5–7)	6 (3–7)	.174*	*r* = 0.198
Toileting	6 (1–7)	6 (5–7)	.687*	*r* = −0.071	6 (5–7)	6 (4–6)	.202*	*r* = 0.186
Bladder control	6 (1–7)	7 (6–7)	.079*	*r* = −0.304	6 (5–7)	5 (3–7)	.374*	*r* = 0.130
Bowel control	6 (1–7)	7 (6–7)	.095*	*r* = −0.288	6 (5–7)	6 (4–7)	.399*	*r* = 0.123
Bed/chair/wheelchair	6 (4–6)	6 (5–7)	.351*	*r* = −0.159	6 (5–7)	6 (5–6)	.426*	*r* = 0.116
Toilet	6 (2–6)	6 (5–6)	.835*	*r* = −0.038	6 (5–6)	6 (5–6)	.313*	*r* = 0.147
Tub/shower	5 (1–5)	5 (5–5)	.569*	*r* = −0.103	5 (1–5)	5 (1–5)	.688*	*r* = 0.059
Walking/wheelchair use	5 (1–6)	5 (4–6)	.478*	*r* = −0.119	6 (1–6)	5 (1–6)	.385*	*r* = 0.127
Stairs	5 (1–5)	5 (2–6)	.194*	*r* = −0.221	5 (1–5)	5 (1–5)	.696*	*r* = −0.057
Comprehension	5 (4–6)	7 (5–7)	.004*	*r* = −0.463	6 (3–7)	6 (4–7)	.824*	*r* = 0.032
Expression	6 (4–7)	7 (5–7)	.057*	*r* = −0.322	7 (4–7)	7 (5–7)	.652*	*r* = 0.066
Social interaction	6 (3–7)	7 (7–7)	.057*	*r* = −0.351	7 (6–7)	7 (5–7)	.970*	*r* = −0.005
Problem solving	3 (2–5)	6 (3–7)	.047*	*r* = −0.325	4 (2–7)	3 (1–5)	.329*	*r* = 0.142
Memory	3 (3–5)	6 (3–7)	.026*	*r* = −0.360	5 (3–7)	4 (1–5)	.049*	*r* = 0.287

Data are presented as mean ± SD, median (IQR), or number (%), as appropriate. Polypharmacy was defined by a median-based cutoff of the number of medications at discharge. The choice between mean or median was based on the results of the Shapiro–Wilk test for normality. Effect size was calculated using Cohen *d* for *t*-test, rank biserial correlation *r* for Mann–Whitney *U* test, and Cramér *V* for categorical variables.

BMI = body mass index, FIM = functional independence measure, IQR = interquartile range, SD = standard deviation.

* *P*-value from Mann–Whitney *U* test.

† *P*-value from independent *t*-test.

‡ *P*-value from Fisher exact test.

### 3.6. Predictors of discharge FIM scores by age: stepwise regression analysis

Table 6 presents the results of the stepwise multiple regression analyses for FIM scores at discharge stratified by age. In the overall sample, the total cognitive FIM score at discharge was significantly predicted by BMI (*β* = 0.284, *P* = .007) and postoperative hospitalization duration (*β* = −0.274, *P* = .010). In the 75–89 years age group, polypharmacy involving ≥10 medications was a significant negative predictor of the total FIM score and the motor subscore (both *β* = −0.398, *P* = .012). For the cognitive FIM subscore, polypharmacy involving ≥9 medications was significantly associated with poorer outcomes (*β* = −0.466, *P* = .003), and this association remained statistically significant after Bonferroni correction. In the ≥90 years group, the total FIM score was negatively predicted by postoperative hospitalization duration (*β* = −0.409, *P* = .004) and the presence of dementia (*β* = −0.288, *P* = .035). For the motor subscore, postoperative hospitalization duration was also a significant negative predictor (*β* = −0.387, *P* = .007). Regarding the cognitive subscore, significant negative predictors included the presence of dementia (*β* = −0.376, *P* = .005), history of fractures (*β* = −0.311, *P* = .019), and the number of days from surgery to admission to the comprehensive rehabilitation ward (*β* = −0.306, *P* = .021). Among these, postoperative hospitalization duration for the total FIM score and the presence of dementia for the cognitive FIM sub-score remained statistically significant after Bonferroni correction.

**Table 6:** Stepwise multiple regression analyses for FIM scores at discharge, stratified by age group.

Discharge	Predictor	*B*	95% CI	*β*	*t*	*P*	VIF	*F*	*P*	Adjusted *R*^2^	Durbin-Watson
Total (n = 86)											
FIM motor	Constant (intercept)	19.017	[10.174 to 27.861]		4.277	<.001		6.230	.003*	0.110	1.982
	BMI	0.564	[0.155 to 0.973]	0.284	2.742	.007	1.027				
	Postoperative to hospitalization	−0.254	[−0.445 to −0.063]	−0.274	−2.639	.010	1.027				
Aged 75–89 (n = 39)											
FIM total	Constant (intercept)	99.355	[89.148 to 109.561]		19.724	<.001		6.966	.012	0.136	2.379
	Polypharmacy (10 or more medications)	−29.355	[−51.891 to −6.819]	−0.398	−2.639	.012	1.000				
FIM motor	Constant (intercept)	71.484	[63.193 to 79.775]		17.469	<.001		6.613	.014	0.136	2.379
	Polypharmacy (10 or more medications)	−29.355	[−51.891 to −6.819]	−0.398	−2.639	.012	1.000				
FIM cognitive	Constant (intercept)	28.852	[26.302 to 31.402]		22.925	<.001		10.263	.003*	0.196	2.289
	Polypharmacy (9 or more medications)	−7.269	[−11.866 to −2.671]	−0.466	−3.204	.003*	1.000				
Aged 90 and above (n = 47)											
FIM total	Constant (intercept)	124.474	[101.401 to 147.547]		10.874	<.001		6.621	.003*	0.196	1.486
	Postoperative to hospitalization	−1.743	[−2.883 to −0.603]	−0.409	−3.081	.004*	1.006				
	Dementia	−24.765	[−47.725 to −1.805]	−0.288	−2.174	.035	1.006				
FIM motor	Constant (intercept)	88.071	[69.423 to 106.719]		9.512	<.001		7.944	.007	0.131	1.728
	Postoperative to hospitalization	−1.312	[−2.249 to −0.374]	−0.387	−2.818	.007	1.000				
FIM cognitive	Constant (intercept)	26.146	[28.764 to 41.168]		11.369	<.001		6.280	.001*	0.256	1.715
	Dementia	−8.723	[−14.709 to −2.737]	−0.376	−2.939	.005*	1.011				
	Fracture	−4.867	[−8.899 to −0.836]	−0.311	−2.435	.019	1.006				
	Postoperative to hospitalization	−0.353	[−0.650 to −0.056]	−0.306	−2.400	.021	1.008				

Stepwise multiple regression was used for model selection. “Postoperative to hospitalization” refers to the number of days from surgery to admission to comprehensive rehabilitation ward.

*B* = unstandardized coefficient, CI = confidence interval, FIM = functional independence measure, VIF = variance inflation factor, *β* = standardized coefficient.

* *P*-values (*P* < .0056) indicate significance after Bonferroni correction for 9 comparisons (3 outcomes × 3 age groups).

## 4. Discussion

This study aimed to explore the association between polypharmacy and ADL in elderly patients, with a novel focus on age group comparisons. We found that hyper-polypharmacy was significantly associated with lower cognitive FIM scores in the 75–89 years group.

Previous studies have reported that in older Japanese outpatients (mean age 76.2 ± 6.8years), a cutoff of ≥5 medications was associated with falls, whereas in older inpatients (mean age 78.7 ± 7.3 years) at university hospitals, a cutoff of ≥6 medications was associated with adverse drug events.^[26,37]^ No significant differences were observed in fall incidence or between age-stratified groups. However, when analyses were conducted using the median number of medications in each age group as the cutoff value, a significant negative correlation was found between cognitive FIM scores, particularly the memory item, and polypharmacy in the 75–89 years age group. Interestingly, in the ≥90 years age group, a positive association was observed between polypharmacy and the memory item of the cognitive FIM score. These findings may have been influenced by several factors commonly observed in older adults, including age-related declines in drug metabolism and excretion,^[38]^ efforts to simplify medication regimens,^[39,40]^ and more cautious treatment approaches for existing comorbidities.^[41,42]^ In addition, differences in disease stage and patient population compared with previous studies may have influenced the results. An increase in the number of prescribed medications due to treatment objectives and changes in general health status, and deprescribing strategies that consider cognitive function in managing drug interactions and side effects may have contributed to these findings.

Negative associations between polypharmacy and physical function, functional ability, cognitive function, and nutritional status have been previously reported.^[43–45]^ The use of 9 to 10 or more medications in the 75–89 years age group was significantly negatively correlated with FIM scores at discharge, consistent with the findings of earlier studies.

The clinical significance of this study lies in the finding that the relationship between polypharmacy and ADL may differ according to age among older adults with proximal femoral fractures admitted to the comprehensive rehabilitation ward. This suggests that, in fall prevention and life support during hospitalization and after discharge, “older adults” should not be treated as a homogeneous group but should receive individualized care based on their backgrounds. Specifically, regarding ADL at discharge, hyper-polypharmacy at discharge was associated with poorer outcomes in the 75–89 years age group and may serve as a potential prognostic factor. In contrast, in the ≥90 years age groups, factors such as the duration required for stabilization after surgery and the presence of dementia, rather than polypharmacy, appeared to contribute to discharge ADL outcomes. However, although statistically significant, the correlation coefficient between the cognitive function assessment scale (FIM) and polypharmacy (*r* = −0.466, *P* < .05) indicates a moderate effect size, suggesting that although there is a clinically meaningful effect, it is not necessarily a large effect.

This study had several limitations. First, this was a single-center, exploratory, retrospective cohort study conducted during the coronavirus disease 2019 pandemic; thus, an adequate sample size could not be obtained. Given the relatively small sample size (n = 86), particularly for stratified analyses, statistical power may have been insufficient to detect more subtle associations. Second, in-hospital falls are rare events that should ideally not occur, and each fall case should have been analyzed detailedly. As many factors are associated with falls, more refined analyses may have been possible by categorizing falls according to factors such as time of occurrence and location. Third, the analysis of medication use in this study was limited to drug categories identified in clinical guidelines as being associated with fall risk. This selection was made to reflect the practical scope of physical therapists, who typically do not possess detailed pharmacological training. We acknowledge that more comprehensive analyses involving drug classification and combination effects would be valuable. However, we also believe that simplified and easily applicable approaches, as adopted in this study, are important for promoting broader use in clinical rehabilitation settings. Future studies should consider collaboration with pharmacists and medical doctors to explore more advanced and detailed medication-based risk stratification. Considering these limitations, future directions should include multicenter, multiregional, prospective observational studies, and intervention studies to obtain higher levels of evidence.

## 5. Conclusion

Among older adults with proximal femoral fractures admitted to a comprehensive rehabilitation ward, a significant association between polypharmacy and FIM scores at discharge was observed in the 75–89 years age group. Additionally, the factors associated with ADL at discharge appeared to differ between the 75–89 and ≥90 years age groups. These findings highlight the importance of individualized approaches considering the patient’s age and background when providing support during hospitalization and after discharge.

## Acknowledgments

We would like to express our sincere gratitude to the patients who participated in this study, as well as to the physicians, nurses, therapists, and all staff of the Rehabilitation Department at Tsurugi Public Hospital for their invaluable support.

## Author contributions

**Conceptualization:** Eisuke Takeshima, Akira Kimura.

**Data curation:** Eisuke Takeshima, Akira Kimura.

**Formal analysis:** Eisuke Takeshima, Akira Kimura.

**Investigation:** Eisuke Takeshima.

**Methodology:** Eisuke Takeshima, Akira Kimura.

**Project administration:** Eisuke Takeshima, Akira Kimura.

**Resources:** Eisuke Takeshima, Akira Kimura.

**Validation:** Eisuke Takeshima, Akira Kimura.

**Visualization:** Eisuke Takeshima.

**Writing – original draft:** Eisuke Takeshima.

**Writing – review & editing:** Eisuke Takeshima, Akira Kimura.

**Supervision:** Akira Kimura.
